# Comparative analysis of three decades' experience in the management of pregnant women with epilepsy: a real-life scenario

**DOI:** 10.3389/fneur.2023.1254214

**Published:** 2023-09-13

**Authors:** Réka Válóczy, István Fekete, László Horváth, Zsófia Mészáros, Klára Fekete

**Affiliations:** ^1^Faculty of Medicine, Doctoral School of Neuroscience, University of Debrecen, Debrecen, Hungary; ^2^Department of Neurology, Faculty of Medicine, University of Debrecen, Debrecen, Hungary; ^3^Department of Pharmaceutical Surveillance and Economy, Faculty of Pharmacy, University of Debrecen, Debrecen, Hungary

**Keywords:** pregnancy, antiseizure medication, seizure, women with epilepsy (WWE), Epilepsy Care

## Abstract

**Introduction:**

People with epilepsy have to face many challenges, including regular follow-ups, the need to take antiseizure medications (ASMs), and the fear of seizures. Pregnant women with epilepsy (PWWE) are a special group with even more challenges because they are responsible for the fetus. We aimed to evaluate the change in the frequency of pregnancies over the years and the possible role of newer types of ASMs concerning this change, the shift in medication use over three decades, and their possible impact on the outcome of the observed pregnancies.

**Methods:**

Data were retrieved from the prospective Epilepsy Database of the Outpatient Clinic at our tertiary center between 1 January 1992 and 31 December 2020. Groups were formed for comparison in time and depending on whether regular care consultation was our task. Statistical analysis was carried out using Microsoft Office Excel 2021. Basic statistics and categorical variables were assessed using Pearson's χ^2^ test with Yates' correction. Differences were considered significant if the *p*-value was <0.05. The odds ratio and 95% confidence intervals were calculated wherever needed.

**Results:**

Altogether, 181 pregnancies were studied, mostly after 2002. The regular follow-up group consisted of 101 patients, with 44.5% presenting in the first trimester. The majority of seizures were either generalized or focal to bilateral tonic-clonic seizure types (85.6%). Pregnancies ended in live births in 91.7%, which gradually improved over time, while spontaneous abortion did not differ significantly in the time interval groups. Mostly, monotherapy was provided. PWWEs had higher chances for seizure freedom in the regular-care group I: OR = 2.9 (2.15–3.65) *p* < 0.0001. A shift toward newer-type ASMs was found as time passed. Levetiracetam and lamotrigine were more commonly used in the regular care group I than by those patients who were sent to consultation only and not treated at our center [OR = 3.18 (2.49–3.87)] *p* < 0.0001.

**Conclusion:**

This is the first study in our region to evaluate experience in the treatment and outcome of PWWE. Having received reliable care and safer ASMs, the number of pregnancies among PWWEs grew. Data suggested that specialized centers' care offered cooperation with obstetricians is important. Moreover, professional care can also enable PWWEs to have uneventful pregnancies.

## Background

The prevalence of epilepsy in the general population is estimated to be between 0.3 and 1%, making it the most common neurological disease. Based on this, there are ~50–60,000 patients with epilepsy in Hungary alone. Considering that the female-male ratio is ~1.1/1.7, this indicates that there are ~30,000 women with epilepsy (WWE) in Hungary (1). This substantial population of patients needs special care and consultation throughout the course of their disease.

The female hormonal cycle can affect seizure frequency and vice versa: epilepsy can contribute to hormonal changes (2). Disorders of fertility are more frequent in the WWE than in the normal population. The hypothalamus-hypophysis axis can be altered both as an adverse reaction to antiseizure medications (ASMs, e.g., VPA) or by the disease itself. Cases of polycystic ovarian syndrome (PCOS) are more often found in temporal lobe epilepsy (2). As for social factors, WWE has to face a social stigma that is still present today. These patients are also exposed to irrational fear due to a lack of proper consultation about the actual risks and the effects seizures and ASMs might have on their fetuses (3).

For most women with active epilepsy, continuing treatments during pregnancy is necessary. However, prenatal exposure to ASMs is associated with increased risks of growth restriction and congenital malformations. More information is needed to support WWE and their physicians in making a well-informed decision regarding medication use during pregnancy, specifically about newer ASMs, as most studies were conducted well-before the newer types were introduced.

It is important to understand that the majority of WWE can have and do have uneventful pregnancies and give birth to healthy children. The seizure burden remains the same during pregnancy for approximately two-thirds of women, and many of them remain seizure-free (4, 5). Despite this, certain risks need to be discussed thoroughly, as both the treatment and the lack of it carry potential harm for the mother and fetus alike. Generalized tonic-clonic seizures (GTCSs) are associated with hypoxia and lactic acidosis, which can lead to fetal asphyxia (6, 7). Seizure-related falls can also cause blunt trauma to the uterus and thus affect the fetus. Uncontrolled seizures also increase maternal morbidity and mortality (8, 9).

Several ASMs have been associated with teratogenic properties. Many studies have shown that the risk is higher with VPA. In a recent study conducted at 21 centers, VPA was associated with a significantly higher risk of congenital malformations, whereas LTG had the weakest association (10). Other older agents, such as phenobarbital (PB) or phenytoin (PHT), have also been shown to have higher risks of teratogenicity compared with newer-type ASMs such as lamotrigine (LTG) and levetiracetam (LEV) (11, 12). Common and potentially adverse fetal effects include intrauterine growth restriction (IUGR), dysmorphisms, major congenital malformations (MCMs), and delays in postnatal cognitive development. Well-known examples are heart defects among children exposed to barbiturates, fetal hydantoin syndrome in those exposed to PHT, neural tube defects, and skeletal abnormalities associated with VPA and carbamazepine (CBZ).

The risk of malformations is two to three times higher than expected in the general population with older-generation drugs. This risk is often observed in a dose-dependent manner. Treatment with more than one drug is also associated with higher rates of birth defects (13–15). As for newer agents, topiramate (TPM) is best known to have teratogenic properties, and while more and more data were collected and analyzed about newer ASMs, they are still incomplete compared to older ones. In the past several years, different registries were created for this patient population to make the data more comprehensible. These registries [e.g., European Registry of Antiepileptic Drugs and Pregnancy (EURAP), UK, and national registries] collect high numbers of pregnancies, the type of drug exposure is recorded in an unbiased way, and other possible confounding factors are also listed (e.g., alcohol use and genetic abnormalities) (16). Based on this massive data collection, studies have demonstrated the relative safety of LTG and LEV, but even more information is needed.

This is the first study in our region to evaluate the experience of treatment and outcome in this fertile patient population. Below, we attempt to evaluate the change in the frequency of pregnancies over the years and the possible role of newer-type ASMs in regard to this change, the shift in medication use over three decades, and their possible impact on the outcome of the observed pregnancies.

## Methods

### Patients, database

Data were retrieved from the prospective Epilepsy Database of the Outpatient Clinic of the Department of Neurology at the University of Debrecen, containing encrypted data of all patients appearing in our Epilepsy Outpatient Clinic for the retrospective analysis of pregnant women with epilepsy (PWWE). The files selected are from the period between 1 January 1992 and 31 December 2020.

The subjects were WWE, managed by a tertiary teaching hospital. Each pregnancy of the same patient was considered a single case. The department provides care only for adult patients. The catchment area was ~548,000 inhabitants.

The criteria for recruiting participants for this study were either a diagnosis of epilepsy and/or the presence of an ICD code (International Statistical Classification of Diseases and Related Health Problems) (17), specifically G40-41, documented in the patient's medical file. Patients with a presumed drug-provoked seizure, psychogenic seizure, or cardiogenic etiology were excluded. Patients undergoing epilepsy surgery were also excluded. If a sole PNES (psychogenic non-epileptic seizure) was suspected, a video EEG was made. In cases where PNES (and not epilepsy) was diagnosed, the data of the patient was deleted from the database.

In every case, data collection the following data: age, age at the onset of epilepsy, seizure type in accordance with the ILAE definitions (18), age at giving birth, family history of epilepsy, ASMs taken before, during, and after pregnancy, amount of ASM doses used, ASM serum levels, seizure frequency before and during pregnancy, other medicines regularly taken for the CNS, MRI scans, EEGs, and co-morbidities.

The ILAE definitions were modified in 2017 (18). In this study, we used the latest version, although most files included here used previously common expressions (e.g., in the records, many women are referred to as having “complex partial” seizures, now referred to as “focal seizures with impaired awareness”, or another example might be “petit mal”, currently called “absence”).

A certified epileptologist diagnosed and provided care for these patients, and the ASM used in each case was prescribed by that specialist.

- Four groups were formed for comparison (time of pregnancy):

G1: Before 1992

G2: 1992–2001

G3: 2002–2011

G4: 2012–2020.

The first group (G1) included patients who reported having pregnancies before 1992, which is the year the epilepsy outpatient unit in our department was established. In the second group (G2), the patients were treated with older-generation ASMs; newer ASMs became available on the market after a millennium (i.e., in Hungary, LTG was allowed to be used during pregnancy around 2002–2003). In the third group (G3), LTG and newer drugs were used more commonly, becoming the drugs of choice in the pregnant population.

- Providing care was another point of view in forming groups for comparison, which is as follows (care groups):

I. Patients with epilepsy (PWE) who were treated at our Epilepsy Care Center
II. Patients who first acquired seizures during pregnancy and stayed in our Epilepsy Care Center
III. Patients were sent only for consultation by their gynecologist.

We collected data about seizure types, the number and type of ASMs used, the outcome of pregnancies, rates of spontaneous and induced abortions, complications during pregnancy, and foetopathies. The results were compared among these three groups.

### Statistics

Statistical analysis was carried out using Microsoft Office Excel 2021. Besides the basic statistics, categorical variables were assessed using Pearson's χ^2^ test with Yates' correction. A *p*-value of <0.05 was considered statistically significant. The odds ratio and 95% confidence intervals were calculated where needed. Ethical approval was obtained from the Regional and Institutional Ethics Committee (DE RKEB/IKEB: 5472-2020).

## Results

### Basic characteristics

In total, we collected data on 191 pregnancies by 127 women (Table 1). After data clearing, 181 pregnancies were included in the database. Ten pregnancies were excluded because of insufficient data and/or because the pregnancies happened years before the onset of epilepsy. Of all the people studied, 33 women had a positive family history of epilepsy (18.2%). Comparing the mean age in the G4 group to the other groups' (G1, G2, and G3), significantly lower ages were detected (*p* < 0.05). If the mean age in care group I was compared to the mean age in group II, it was significantly lower (*p* = 0.002).

**Table 1:** Basic characteristics of the studied groups.

	**G1 < 1992**	**G2 1992–2001**	**G3 2002–2011**	**G4 2012–2020**	**I**.	**II**.	**III**.	**Total**
No. of pregnancies	10	28	65	78	101	13	67	181
Average age at pregnancy (years ± SD)	18.3 ± 3.7^*^	26.5 ± 6^*^	28.7 ± 4.7^*^	30.9 ± 5.8	29.6 ± 5.3	24.7 ± 4.2^*^	28.4 ± 6.7	28.76 ± 6

* *p* < 0.05.

**Figure 1:**
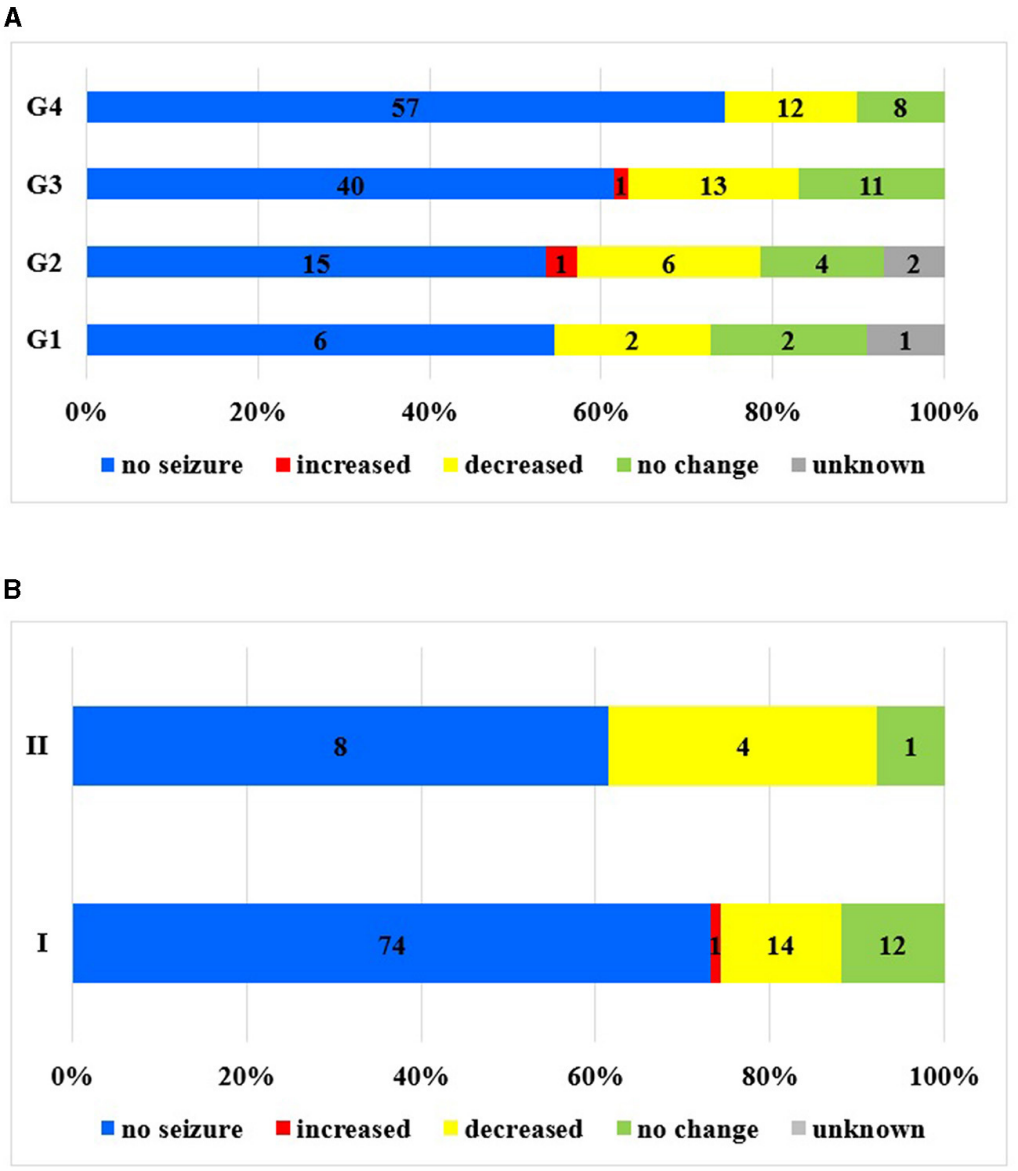
Seizure frequency changes in the examined periods. **(A)** Seizure frequency changes during pregnancy in different time intervals. **(B)** Seizure frequency changes during pregnancy in the different care groups I–II.

The number of women becoming pregnant and the number of pregnancies and births have kept growing since the 2000s, and even though it did not reach statistical significance, a trend is clearly evident (Figure 1). Of all examined pregnancies, 79% happened after 2002. Nevertheless, the number of new patients also grew steadily: (all new female patients) 153–47.7%, 497–45.7%, 877–53.2%, and 1,112–55%), in the time period of <1992, 1992–2001, 2002–2011, and 2012–2020, respectively.

In group I, 44.5 and 25.7% of the pregnant women presented in the first and second trimesters, respectively. Over time, more PWWE arrived at an earlier stage of pregnancy; a strong, increasing trend in their number could be detected. Patients referred by gynecologists, those treated at other hospitals, or those without regular follow-ups were evenly distributed across different trimesters. Patients in group II, 61.5% (*N* = 8), appeared mainly in the second and third trimesters since toxemia or cerebrovascular complications develop more frequently in these trimesters.

### Seizure types

More than half of the patients (*N* = 104, 57.4%) experienced generalized seizures, with tonic-clonic being the most common (a total of 56.9%). The remaining women had other types of seizures, such as absence, myoclonic, or atonic seizures. An additional 28.2% (*N* = 51) of the subjects had focal to bilateral tonic-clonic seizures. In the studied population, 41.9% (28.2% focal to bilateral tonic-clonic seizure, 13.7%) had a focal seizure. No significant difference was observed concerning the seizure types between the observed periods. Of all pregnancies in our study (*N* = 181), EEG recordings were made in 69% (*N* = 125), and 11.2% (*N* = 14) of them showed interictal epileptiform abnormalities. The distribution of EEG tests in the different care groups was as follows: group I, 75.2% (*N* = 76); group II, 46.2% (*N* = 6); and group III, 64.2% (*N* = 43). Comparing the time interval periods, the distribution of EEG recordings was the following: 100% (*N* = 10) in G1, 57.1% (*N* = 16) in G2, 66.2% (*N* = 43) in G3, and 71.8% (*N* = 56) in G4.

There were no seizures during pregnancy in two-thirds of the patients (65.2%, *N* = 118).

If different time intervals were examined, the ratio was even better: G1, 54.5%; G2, 53.6%; G3, 61.15%; and G4, 86%. Hence, seizure freedom has become more common over the past decades. If the follow-ups (I) were carried out in our tertiary center, the ratio of seizure freedom during pregnancy was 73.1%, while in the consultation group (III), it was 55.2% (Figure 1). An increase was most often noted in the second trimester (50% of known cases), followed by the third (37.5%) and first trimesters (12.5%).

Due to maternal non-compliance, seizures in the peripartum period were detected in all pregnancies as follows: G2, four cases (14%); G3, four cases (6.2%); and G4, five cases (6.4%). In G2 and G3, three out of four (75%) and four out of five (80%) pregnancies, respectively, were carried out with a previous seizure-free period of more than 12 months. In G4, all five pregnancies (100%) were seizure-free.

In the case of care group I, 10 cases (9.9%) were detected. Seizure freedom could be observed in 73.3% of the pregnancies. No change in frequency was observed in 11.9% of the cases, while in 13.8% of the cases, the frequency of seizures decreased, and seizure frequency increased in one patient (0.9%) only.

During pregnancy, in a total of 37 cases (20.4%), a tonic-clonic seizure was recorded. The percentage of cases with one seizure kept decreasing in the studied periods: G1, 50% (*N* = 5); G2, 17.8% (*N* = 5); G3, 21.5% (*N* = 14); and G4, 16.6% (*N* = 13). Examining the groups categorized according to the type of care, we found the lowest seizure frequency in pregnancies where regular care was provided in a tertiary center (I. 12.8% (*N* = 13), II. 46.1% (*N* = 6), III. 26.8 % (*N* = 18) (*p* = 0.005). A PWWE had a higher chance of having seizure freedom in the regular-care group I: OR = 2.9 (2.15–3.65) *p* < 0.0001.

### Treatment

Data collection started in 1992 when newer types of ASMs were not yet commercialized: e.g., LTG was first launched in 1994 and allowed to be used during pregnancy only after 2002. Its use became widespread in the 2010s.

**Figure 2:**
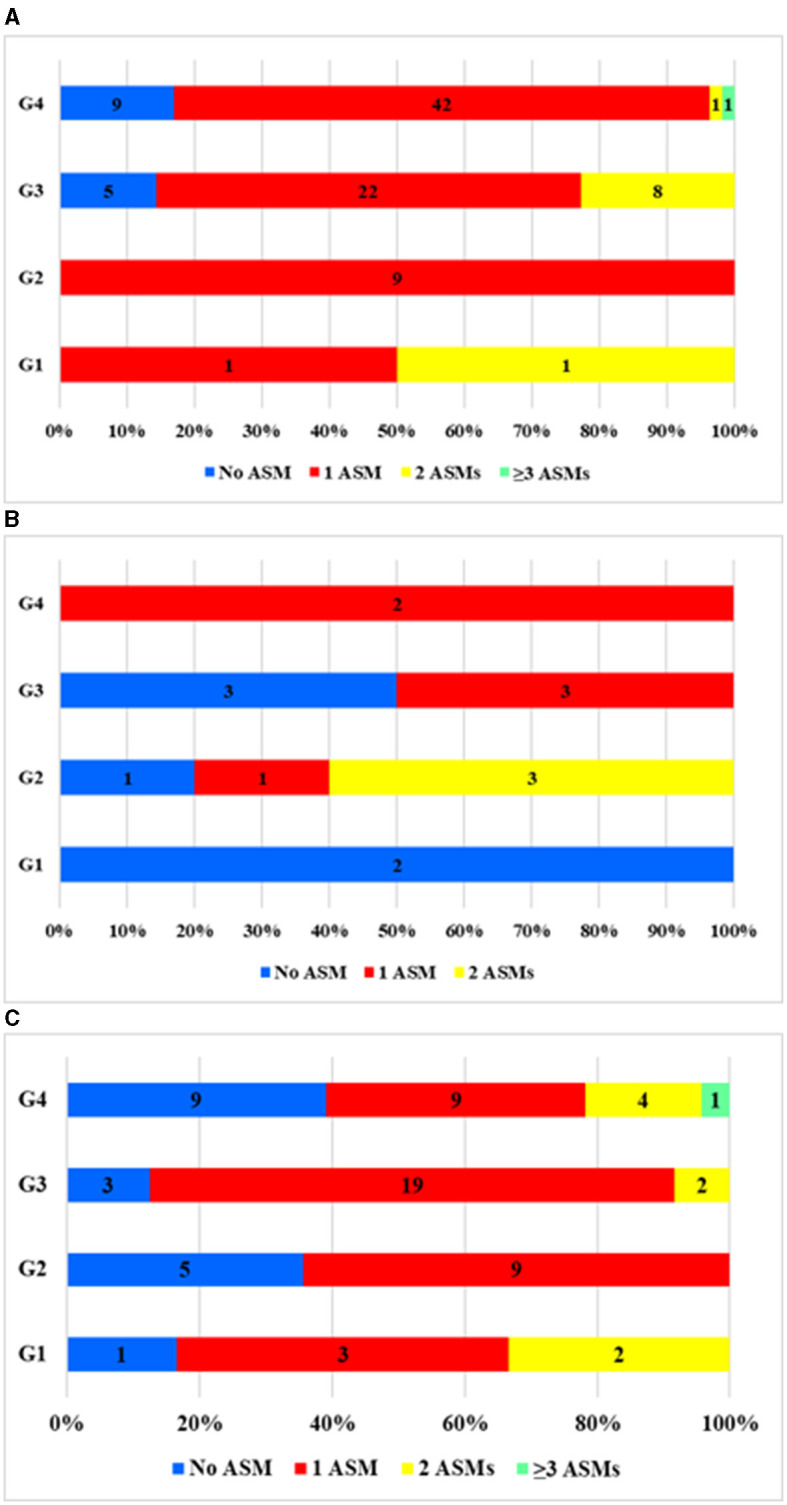
Distribution of pregnancies by number of ASMs taken during pregnancy by the examined period (G1, 2, 3, and 4) in each care group (I, II, and III). **(A)** Care group I (*N* = 101). **(B)** Care group II (*N* = 13). **(C)** Care group III (*N* = 67). No ASM, the patient did not take any ASM; 1ASM, monotherapy; 2ASM, biotherapy; ≥3 ASM, polytherapy during pregnancy.

**Figure 3:**
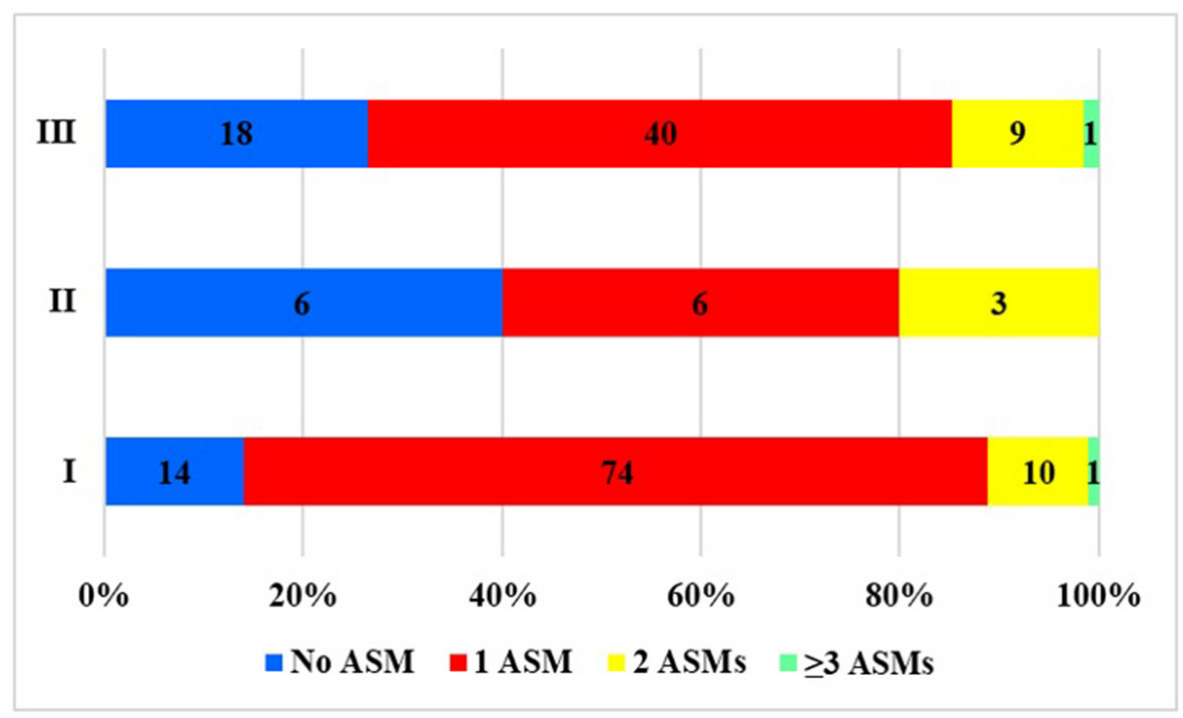
Distribution of pregnancies by the number of ASMs taken during pregnancy based on the type of care. No ASM, the patient did not take any ASM; 1ASM, monotherapy; 2ASM, biotherapy; ≥3 ASM, polytherapy during pregnancy.

In each time interval, different populations were treatment-free: G1, 30% (*N* = 3); G2, 21.6% (*N* = 6); G3, 16.9% (*N* = 11); G4, 23% (*N* = 18). Monotherapy was most common in every time interval (G1-G4) and treatment group (I–III) (Figures 2, 3). If bitherapy was necessary, the typical approach involved combining an older-type ASM with the newer LTG.

**Figure 4:**
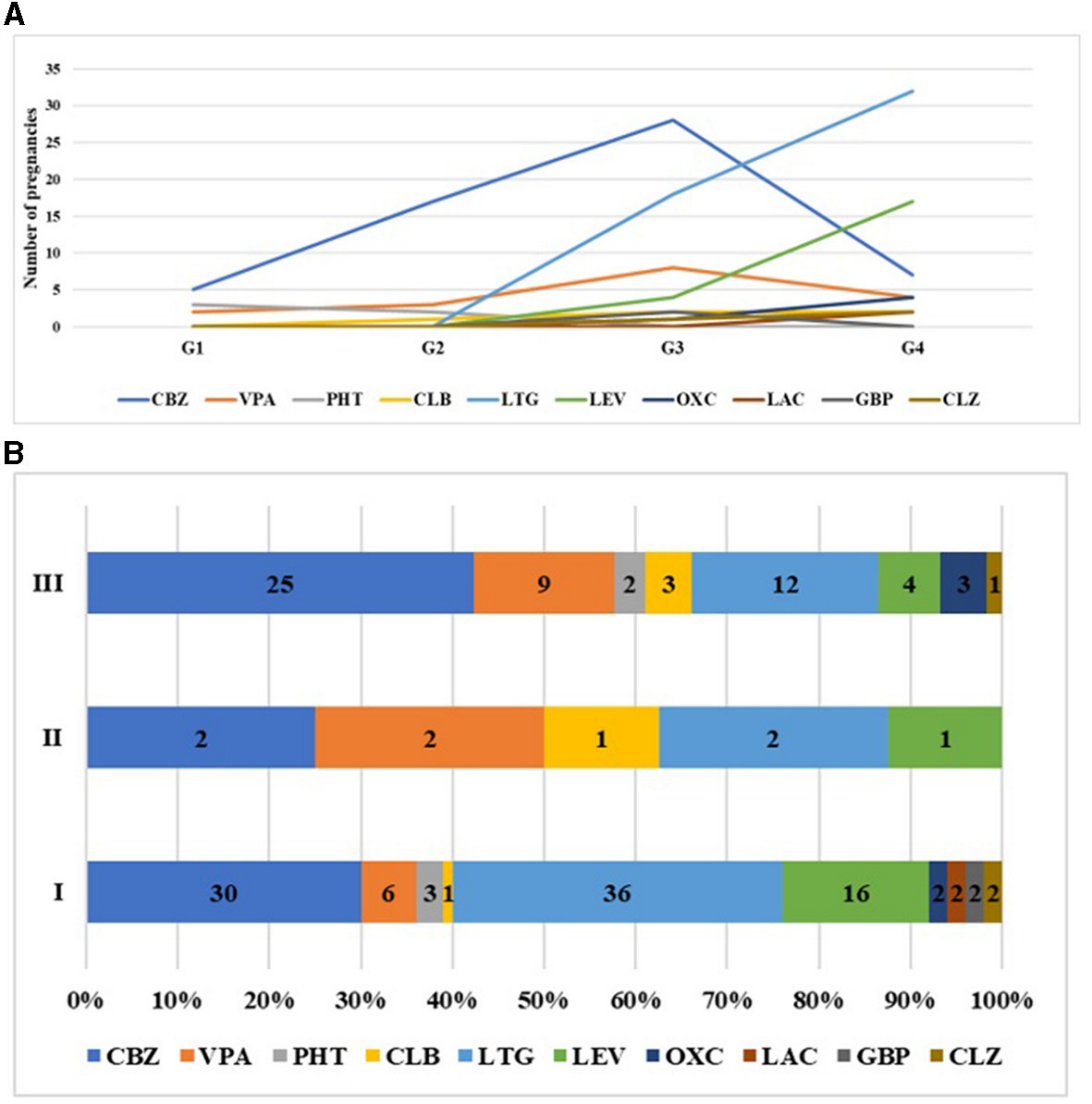
**(A)** Trends of prescribed ASMs during pregnancy between the time interval groups. **(B)** The change in the type of ASMs taken during pregnancy in the different care groups.

When we examined the types of medications (Figure 4A), we found a shift toward newer types of ASMs as time passed. Between 1992 and 2001, only older types of ASMs were in use. After 2002 and, especially after 2012, the use of newer-type ASMs became increasingly common, mainly favoring LTG and, later, LEV. Having become available, ASMs such as lacosamide (LAC), zonisamide (ZNS), and topiramate (TPM) were also prescribed occasionally after 2012. The reduction in the use of CBZ and the rise in the use of LTG were both statistically significant, *p* < 0.0001 (Figure 4A). If the treatment groups were compared, more ASM types and newer ASM types were used (Figure 4B). In group II, the number of patients was too low to draw conclusions. LEV and LTG were more commonly used in the regular care group I compared to patients who were sent to our center for consultation but not treatment (OR = 3.18 (2.49–3.87) *p* < 0.0001.

As for dosage change, treatment group I could be followed clearly. Altogether, the dosage was changed in 39 cases (38.6%). This often meant an increase in the dose taken (94%).

### Outcome of pregnancies

**Figure 5:**
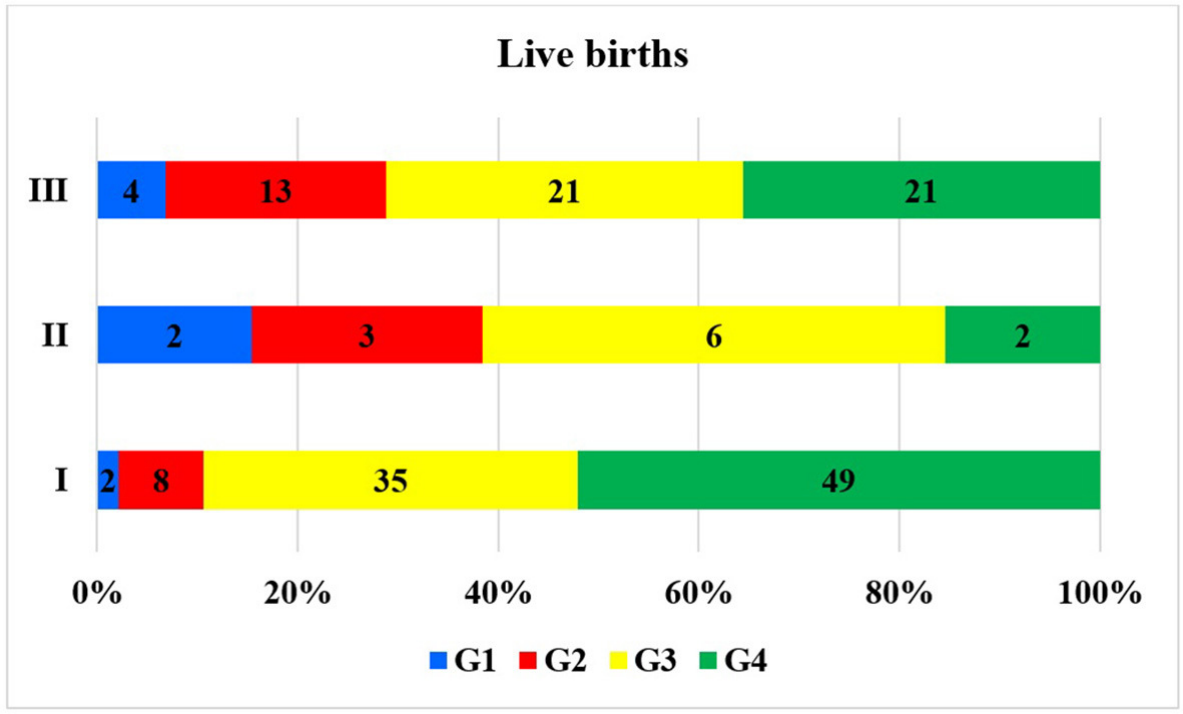
Live births by care groups in different time periods.

**Figure 6:**
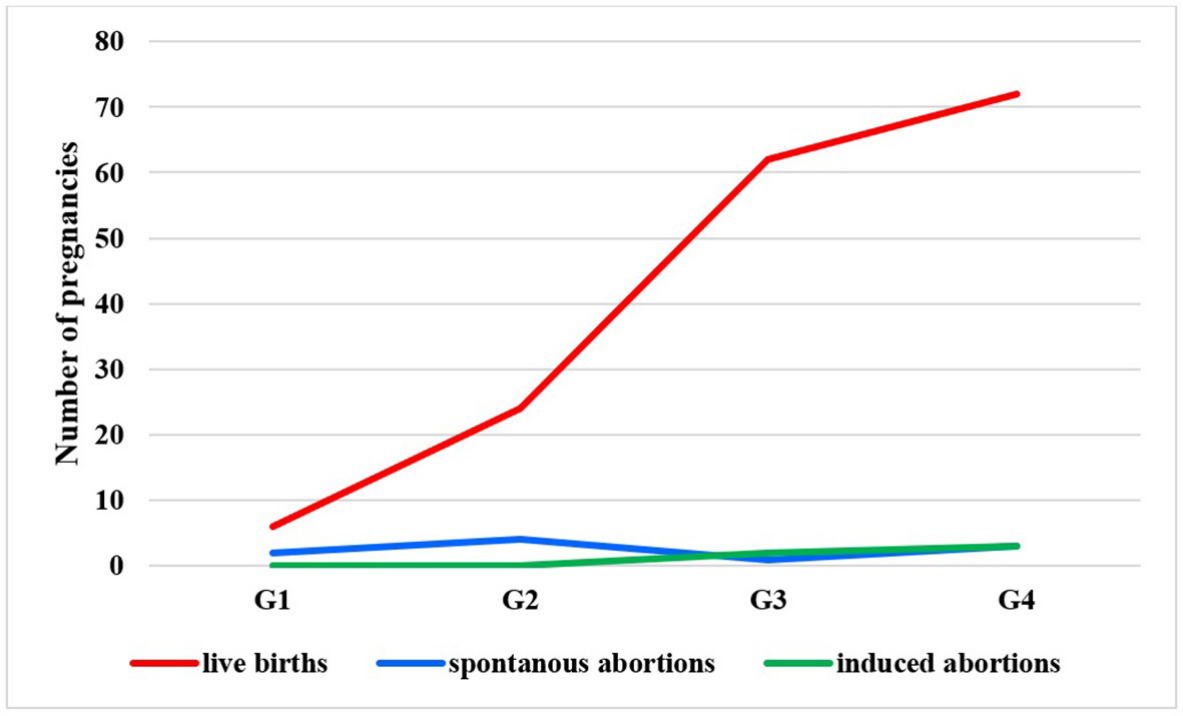
Outcome of pregnancies in the different care groups.

The majority of pregnancies ended with live births (91.7% *N* = 166), which increased over time in each treatment group (Figures 5, 6). Spontaneous abortion did not differ significantly among the time interval groups (5%, *N* = 10), and induced abortion was also low (2.7%, *N* = 5). Over time, the need for consultation by gynecologists increased after 2002.

In most cases, the cause of spontaneous abortion could not be determined. The obstetric examination and, if there were any genetic examinations, the findings were negative.

There was one case (Table 2, *P1*) where the twins had no heart sign on regular examinations in the first trimester.

**Table 2:** Details of adverse outcomes.

**Patient**	**Circumstances**	**Finding in the fetus**	**Outcome**	**Care group**	**Time interval**
P1	Twin pregnancy	Lack of heart signs in the first trimester	Fetal death	I.	G4
P2	Psychiatric therapy, suicide, non-compliance, and 43 years old	Polyhydramnios	i.a.	I.	G4
P3	40 years old and no ASM	None	i.a.	I.	G4
P4	Psychiatric disorder	None	i.a.	III.	G4
P5	Psychiatric disorder	None	i.a.	III.	G4
P6	Tuberous sclerosis	Not reported	i.a.	III.	G3
P7	No ASM	Complex foetopathy	Live birth	III.	G4

i.a., induced abortion.

In cases of *induced abortion* (*N* = 5), two pregnancies occurred in the regular follow-up group (I.), and three pregnancies happened in group III (consultation). The first patient in group I (Table 2, *P2*) was 43 years old and was undergoing psychiatric treatment with multiple medications. She took her own life during her pregnancy, unaware of her condition. Although it is unclear whether she was consistently taking antiseizure medication, the fetus exhibited polyhydramnios. However, subsequent genetic and pathological examinations did not reveal any abnormalities.

The second woman (Table 2, *P3*) was 40 years old. She did not take ASM and had no seizures during her pregnancy. In group III, two women (Table 2, *P4, P5*) received treatment for psychiatric diseases, and another one was included because of tuberous sclerosis (Table 2, *P6*).

Besides the foetopathies mentioned earlier, one pregnancy exhibited complex foetopathies (multicystic kidney, pyelectasis, macrosomia, and polyhydramnios). The patient who experienced epilepsy but was not taking antiseizure medication during her pregnancy was initially referred to us for consultation and remained under our care thereafter (Table 2, *P7*).

Thus, no direct association could be established with epilepsy. A more detailed neonatological examination would have revealed additional abnormalities. Altogether, some kind of foetopathy was mentioned in six pregnancies (3.3%). In one case, the patient was on CBZ; in another, she used LTG; and in the third case, it is uncertain whether the mother took any antiseizure medication (ASM) (as previously mentioned). The remaining pregnancies proceeded without the use of ASM.

Most pregnancies were unplanned without problems (86.7% *N* = 157). Six of the pregnancies (3.3%) had complications during delivery, mostly of gynecological origin (e.g., placental abruption, meconium, or preterm delivery). One of the patients suspended from taking ASM in the third trimester had a seizure; consequently, her gynecologist had to perform a cesarean section. Five of the patients had toxemia, eclampsia, and some had seizures after delivery and stayed in our care. One patient developed herpes encephalitis and had a middle cerebral artery occlusion with symptomatic seizures. These patients were not in our care (groups II., III.).

## Discussion

The treatment and guidance of pregnant epileptic patients involve several considerations in balancing the risks between the mother and her child. Care for pregnant women with epilepsy requires a specialized team, early advice, and coaching to make the best and safest plan possible for both the woman and her child (19). In the past decades, to provide evidence-based care for them, many countries and different registries have started collecting and analyzing data to fulfill their task (20). To our knowledge, our study is the first to collect such data and address this issue in Hungary.

In the 20th century, WWE often hesitated to risk becoming pregnant due to potentially severe impacts on their quality of life. Even when they did become pregnant, they had to face the possibility of either inadequate seizure control or the devastating risk of fetal complications (20). As safer drugs became available, WWE tended to have more and more pregnancies carried out while using ASMs. Besides, the number of new patients also grew gradually, as did the ratio of WWE among them. More factors were responsible for more women turning to our center for care individually or at their gynecologist's request. Epilepsy became more prominent in our region due to health education, and regular consultations with gynecologists also facilitated positive changes. Our study supports this finding: more than 80% of the studied pregnancies happened after 2002. A Danish study found similar results. The proportion of pregnant epileptic patients rose from 0.5% in 1989–1993 to 0.98% in 2009–13, according to their study (21).

Moreover, in a Canadian study, the number of pregnancies increased gradually (22). Over time, women were older at the time of becoming pregnant. This might be because more WWEs had more than one child, so they were older when they became pregnant again. In addition, women's ages during pregnancy also increased in the population. Similar results were found in a study by Shihman et al. (23).

Our results align with the general age trends for women becoming pregnant in the Hungarian population (24). Over the years, many more patients have had regular checkups (I) earlier in the first trimester, emphasizing the importance of care, which was also recognized by patients. Sometimes, the first epileptic seizures may be experienced during pregnancy, especially due to toxemia or diseases of the central nervous system. These cases also require attention since these patients are unfamiliar with epilepsy, so their examination and follow-up are important. The result of care group III highlights the importance of tertiary epilepsy centers where gynecologists can consult with specialists having experience with PWWE.

Among the seizure types, tonic-clonic seizures pose the greatest risk to the fetus the most, regardless of whether they are generalized or focal-to-bilateral tonic-clonic seizures. In our study, these were present in more than three-quarters of the pregnancies (85.6%) and were significantly less common in the regular care group I. This emphasizes the importance of regular checkups and good cooperation with the patient. Peripartum seizures occurred in our follow-up group I due to poor maternal compliance. This was often driven by fears of fetal abnormalities and, in some cases, was due to alcoholism. Our data indicate that most of our patients underwent EEGs, which revealed epileptiform activity that informed adjustments in treatment. As a result of our findings, we offered every pregnant woman to do an EEG in the second trimester and, if necessary, to repeat it for over a decade.

Monotherapy was the most common choice for treatment, chosen for its effectiveness and safety. We found different patterns in the different treatment groups. Only a few patients received more than two ASMs. Biotherapy was especially useful when LTG or LEV alone was not enough. As part of bitherapy, ASM (e.g., CBZ and VPA) could be administered at lower doses. When discontinuing VPA was not an option, although the guidelines for VPA in fertile ages came out later, the dosage of VPA was kept as low as possible (mostly under 600 mg) in all-time groups.

It might be assumed that having access to the newer types of ASMs, women were more inclined to undertake pregnancies, even when taking more medications. This could be a contributing factor to the growing number of pregnancies observed. Moreover, better compliance was probably an important cause; as safer ASMs became available, more women took their ASMs and experienced no seizures. Although seizures still occurred in some cases, the frequency increased only in a few cases.

Major changes in the favored drugs of choice for use in pregnant epileptic patients have been observed over the years. While carbamazepine was the number-one choice in the 1990s, its use showed a huge decline after the 2000s. Simultaneously, lamotrigine had a steep rise, followed by levetiracetam in the 2010s. Similar findings were also found after the analysis of the EURAP registry (16, 25). The use of LTG and LEV gradually increased over the years, especially in the regular care group I, where the choice of ASM favored medications with fewer or no side effects for pregnancies (26). Unfortunately, there were some patients, even in the regular care group I, in whom VPA could not be discontinued, but the dosage could be reduced (<600 mg). Although the effects of VPA on the fetus have been discussed in a recent publication (10), a study from 1999 reported that VPA might have a negative impact on pregnancy in a dose-dependent manner (15). Therefore, we decreased the dose of VPA as much as we could to reduce the risk of side effects already in the early treatment groups (even in G2). According to the literature, exposure of the fetus to the new ASMs is unlikely to increase the risk of major congenital malformations compared to older drugs (27). Therefore, the importance of regular care with the use of LEV and LTG can be emphasized, which is more common in this group, showing that in this case, the epileptologist will consider a safer ASM in fertile women in due time (25).

Among our patients, few had to take psychiatric medications during pregnancy or had severe psychiatric problems, which might increase the risk of abortions and foetopathies. According to Bangar et al. (28), these women have to cope with two clinically relevant and important issues simultaneously, besides possible drug interactions and non-adherence, emphasizing the importance of care.

Dosage changes usually mean an increase in a quarter of all patients. The factors associated with dosage change were a low serum ASM level or interictal epileptiform discharges on EEG. Low serum ASM levels were most often observed with the use of LTG. The rest were associated with LEV or CBZ. It is well-known that some ASDs are likely to have a decreased serum level due to physiological changes during pregnancy (29). Our results are comparable to those based on data from the EURAP registry. In this study, doses had to be increased in 26% of the cases. We also found that lamotrigine needed to be increased most often, followed by levetiracetam and valproate.

In our study, most pregnancies in WWE were uneventful and could be carried out without major problems. For most women, if they had seizures at all, they remained focal, and even if they had tonic-clonic seizures, generally associated with worse outcomes, the actual burden was small. All these findings concord with the findings from several different registries, like the EURAP and Danish registries, to mention but a few (5, 16, 21).

WWE's live births were 91.7%. As a matter of fact, 91.7% of the WWE in this study delivered live babies; this finding is similar to that from a Turkish study (10). Remarkably, more pregnancies ended with live births over time. The rate of spontaneous and induced abortions was relatively constant. Similar to data from the Hungarian Central Statistical Office, the rate of spontaneous abortions remained between 7 and 8/1,000 women from the 1990s until now in the general population.^1^ Abortions were mainly miscarriages. Some studies link older-type ASM use with higher rates of miscarriages (30), but these findings could not be confirmed by many larger cohorts. A study observed almost a million pregnancies where the spontaneous abortion rate did not increase among WWE-taking ASDs compared to those who did not (31). Unfortunately, fetal death happened in a twin pregnancy, which means 0.5%, whereas, in a meta-analysis, stillbirth and fetal death represented 1% (32).

The study based on the EURAP registry found no differences in the intrauterine death rates between old and new monotherapies. An increased risk was only found with polytherapy (33). In our study, we did not find a relationship between spontaneous abortions and different drugs, nor did we see a higher abortion rate with polytherapy, but we should remark that we had only a few patients on polytherapy. However, some studies assume that polytherapy alone might not be associated with an increased risk, only when the combination contains valproate (34, 35). It is also well-known that fetal complications are dose-related (36). We found no adverse associations with valproate, but the dosage remained low even in the early periods. Although valproate was officially advised against only in 2018, its adverse effects had been published much earlier (37).

For this reason, it has been common practice at our center to avoid prescribing valproate to fertile women since as early as the first few years of the examined period, and ever since, valproate has been used only when necessary. This is represented in our study, as only a handful of the studied WWE were on valproate therapy, most of them at lower doses (<600 mg/day). We also registered very few cases of foetopathies (2.7%), which falls in the range of 2–3% found in the normal population (38). We could not detect any relationship between foetopathies and ASM either. However, we found that patients who received psychiatric treatment, or those who would need it, were at a higher risk for induced abortions.

Overall, regular care is safer for a PWWE, but it is equally important that gynecologists and epileptologists consult about the cases even if the PWWE is treated at another center. Even though their number became lower and lower in our study, there are still women who do not receive the care and information they need. Richmond et al. reported that PWWE is not at higher risk for obstetrical complications in cases of good care (39).

As a result of our data's interim monitoring, the following model was used to take care of PWWE in our center: therapeutic drug monitoring of ASM serum levels in the first and second trimesters and EEG recording in the second trimester. The epileptologist declares the safety of childbirth and lactation from a neurological point of view. Mothers are requested to come for checkups at the end of the postpartum period and regularly during the first year after labor. The child(ren)'s documents during that time are also required. We initiate discussions about pregnancy with women of childbearing age before the pregnancy actually occurs. Over time, fathers come more and more often to our care to support their partners. In the future, we have to be prepared for new aspects of the care of PWWE. As neuromodulation therapy (e.g., DBS) becomes increasingly common for WWE who are drug-resistant epilepsy, pregnancies will probably occur more often. Therefore, the focus will not only be on ASMs but also be on neuromodulation therapies. Recently, a review was published on the safety of deep brain stimulation (DBS) in pregnant women, not only for those with epilepsy but also for those with dystonia and Parkinson's disease (40). Although the review included only four PWWE, the findings appear promising (40–43).

## Limitations

Our study has some limitations. First, the current study is observational. The study included 191 pregnancies, representing a small sample size, which might influence the number of malformations detected. Nevertheless, a real-life scenario has a similar population (23), depending on the catchment area. Second, treatment options and definitions have changed during the investigation period.

Nevertheless, this study has useful assets, including prospective data collection and detailed information on all subjects. A further strength of our study may be the real-life data sets that lead to a better understanding of real-life clinical settings. The single-center scenario for 30 years is also a strength of the current study.

In summary, with reliable, flexible care and safer ASMs, the number of pregnancies has grown over time. We hope that the attitude toward epilepsy is also changing. Based on our data, better care may be provided in specialized centers, but the importance of cooperation with obstetricians is also emphasized. Our data prove that PWWE can also have an uneventful pregnancy with a similar chance if professional care is readily accessible.

## Data availability statement

The raw data supporting the conclusions of this article will be made available by the authors, without undue reservation.

## Ethics statement

The studies involving humans were approved by Regional and Institutional Ethics Committee University of Debrecen, Nagyerdei krt. 98. 4032 Hungary. The studies were conducted in accordance with the local legislation and institutional requirements. Written informed consent for participation was not required from the participants or the participants' legal guardians/next of kin because the data was encrypted, and it was a non-interventional study.

## Author contributions

RV: Conceptualization, Data curation, Formal analysis, Investigation, Visualization, Writing—original draft. IF: Conceptualization, Investigation, Supervision, Writing—review and editing. LH: Conceptualization, Data curation, Formal analysis, Investigation, Methodology, Visualization, Writing—original draft, Writing—review and editing. ZM: Data curation, Writing—review and editing. KF: Conceptualization, Data curation, Formal analysis, Investigation, Methodology, Project administration, Supervision, Visualization, Writing—original draft, Writing—review and editing.

## Footnotes

^1^Központi Statisztikai Hivatal (2023). Available from: https://www.ksh.hu/stadat.
